# Practical Notes and Formulæ

**Published:** 1884-12-20

**Authors:** 


### PRACTICAL NOTES AND FORMULÆ.

**Gonorrhœa Easily Cured.**—Dr. Dellenbaugh, in a communication to the College Clinical Record, says: The injection I have used in cases of acute and subacute gonorrhœa, for more than a year, with the most gratifying results, especially to the patients, who have recovered from two to seven days, is the following:

| | | |
|---|---|---|
| ℞. | Rescorcin | ʒ j |
| | Acid boracic | gr. xx |
| | Zinci acetatis | gr. $\frac{1}{4}$–$\frac{1}{2}$ |
| | Aqua distillat | ℥ iv. M. |

Of this solution two teaspoonfuls are injected three times daily. The germicides, resorcine, and boracic acid are so slightly astringent that it requires the additional zinc salt to restore capillary tonicity. The injection is quite or nearly painless.

In the treatment of the later stages of acute and chronic gonorrhœa, without stricture or granuloma as a complicating factor, I have had the happiest results follow the use of the following injection:

| | | |
|---|---|---|
| ℞. | Hydrarg. chlor. corrosivi | gr. $\frac{1}{4}$–ss |
| | Zinci chloridi | gr. ss–j |
| | Aqua distillat | ℥ viij. M. |

Sig.: A tablespoonful to be injected well down in the urethra three times daily.—*Medical Herald.*

**Boils.**—Dr. John Aulde, following the suggestion of Dr. Sidney Ringer, has met with most satisfactory results by adopting the following plan. The diet is to be regulated, and, if constipation exists, a teaspoonful of magnesia sulph. in a glass of cold water should be taken every morning before breakfast:

| | | |
|---|---|---|
| ℞. | Calcii sulphidi | grs. iij |
| | Sacch. lactis | grs. xxx. |

Misce bene et div. in chart No. xxx.

Sig.: Five powders daily at intervals, between meals.

By this method beginning boils will be aborted, and those far enough advanced to threaten a siege of several weeks and successive crops will soften and heal in such short time that the patient will be surprised at the result. When they can be obtained, granules containing one-tenth grain are to be preferred to the powders. The urine should be examined for sugar, as boils and diabetes often go together.—*Summary.*

**Golden Eye Water.**—

| | | |
|---|---|---|
| ℞ | Sulphate of hydrastia | grs. ij |
| | Distilled water | ℥ j. |

Make solution.

This is an excellent wash for inflamed and granulated lids.—*Med. World.*

**Picrate of Ammonia in Whooping Cough.**—

℞. Ammonia picratis........................grs. ij–iij

Ammon muriat } aa..........................ʒ j

Ex. glycyrrhyz }

Aqua ad...................................fl. oz. iij.

Sig.: Fl. ʒj. every three hours.

Says a writer in the Medical and Surgical Reporter: I have been less disappointed in this treatment than any I have as yet tried. It would seem worth a thorough trial by the profession.

**Enlarged Tonsils.**—Dr. Douglass (in New York Academy of Medicine) said: He would not always resort to the knife for the removal of enlarged tonsil; it could be slowly but surely reduced in size by the persistent use of a powder composed of salt, camphor, ammonia, and sugar of milk.

**Treatment of Acute Alcoholism.**—

℞. Tr. opii deod, }

Ext. hyoscym, }

Chloral hydrat, } aa..........................ʒ j

Pot. bromide, }

Tr. capsici...................................℥ ss

Tr. aconiti rad...............................♏ v

Aq. menth pip. ad.............................℥ iv.

M. Sig.: Two tablespoonfuls, and repeat in four hours if sleep is not produced.—*Medical Annals.*

**A Formula for Nervous Headache.**—Dr. A. L. Hodgdon, of Farmwell, Va., recommends the following recipe for nervous headache:

℞ Alcohol dilut...............................℥ iv

Olei cinnamom.................................♏ iv

Potass. bromid................................ʒ v

Extr. hyoscyam. fl............................ʒ iss.

S. One to two teaspoonfuls, as required.

Dr. Hodgdon has used this combination with universal success. It is not disagreeable to take, and has no bad effects.—*Maryland Medical Journal.*

**Osgood's Cholagogue, or Celebrated Ague Cure.**—

℞ Sulph. quinine...............................ʒ ij

Fluid ext. leptandrin.........................ʒ ij

Saturated tinc. stillingia....................℥ iv

Fluid ext. podophyllin........................ʒ iij

Oil of sassafras..............................drops x

Oil of wintergreen............................drops x

New Orleans molasses, sufficient to make 8 ounces.

M. Dose, one to two teaspoonfuls.—*Med. World.*

**Fahnestock's Vermifuge.**—This is said to be similar to the patent preparation:

| | | |
|---|---|---|
| ℞ | Castor oil | parts 48 |
| | Oil, wormseed | " 48 |
| | Oil, anise | " 24 |
| | Oil, turpentine | " 1 |
| | Tinct. myrrh | " 3 |

Mix.—*National Druggist.*

**Prescription for Dyspepsia.**—The following will be found excellent in cases of dyspepsia, either chronic or acute:

| | | |
|---|---|---|
| ℞ | Elix. pepsin and wafer ash | ℥ iss |
| | Bismuth sub nit | ʒ j |
| | Fl. Ext. hydrast. canadensis | ʒ iss. |
| | Tr. lavender co. } Syrup simplex, } equal parts, q. s. ad | ℥ ij, |

M. Sig. Teaspoonful three times a day before meals.—*Writer in Med. World.*

**In Threatened Abortion** we have frequently used half drachm doses of the fluid extract of conium with the best possible results as regards the prevention of this untoward event.—*Amer. Med. Digest.*

**For Nose-Bleeding.**—The very best treatment for most cases of nose-bleeding is a saturated solution of common salt in water, used with the usual irrigator. Put the nozzle into the nostril that the blood does not come from, and continue until the bleeding stops, which will be in a few minutes. It does not hurt in the least. If salt water does not succeed in stopping the bleeding, use a solution of subsulphate of iron in same manner. I have used them both, and have never known them to fail.—*N. L. Folsom, in Medical World.*

**Cholera Investigations.**—A recent report of the French Commission for investigating the nature of cholera in Marseilles, shows an entire failure to produce cholera in rabbits or other animals by a great variety of experiments with the comma-bacilli, both as found in the intestines of cholera patients and by cultivation, and, consequently, it affirms the non-contagiousness of that disease.

The report describes a peculiar condition of many of the blood-corpuscles, which the commission regard as the essential pathological condition in epidemic cholera.—*Jour. Am. Med. Association.*

**Simple Means of Obtaining Local Anasthesia.**—Dr. Cheize writes, that wishing to remove an ingrowing toe-nail, and being without a spray-producer, he covered the toe with a pledget the size of a crown piece, poured ether on it and evaporated this by means of a pair of bellows ; in five minutes anesthesia was complete and lasted while the nail was removed and the matrix seared with the actual cautery.—*Glasgow Med. Jour.*

